# Mutations and clinical characteristics of dRTA caused by *SLC4A1* mutations: Analysis based on published patients

**DOI:** 10.3389/fped.2023.1077120

**Published:** 2023-01-26

**Authors:** Mengge Yang, Qiqi Sheng, Shenghui Ge, Xinxin Song, Jianjun Dong, Congcong Guo, Lin Liao

**Affiliations:** ^1^ Department of Endocrinology and Metabology, The First Affiliated Hospital of Shandong First Medical University & Shandong Provincial Qianfoshan Hospital, Ji-nan, China; ^2^ Cheeloo College of Medicine, Shandong University, Department of Endocrinology and Metabology, Shandong Provincial Qianfoshan Hospital, Shandong Key Laboratory of Rheumatic Disease and Translational Medicine, Shandong Institute of Nephrology, Ji-nan, China; ^3^ Division of Endocrinology, Department of Internal Medicine, Qilu Hospital of Shandong University, Ji-nan, China; ^4^ College of Traditional Chinese Medicine, Shandong University of Traditional Chinese Medicine, Ji-nan, China

**Keywords:** distal renal tubular acidosis, *SLC4A1*, metabolic acidosis, mutation, clinical characteristics

## Abstract

**Background and Aims:**

The genetic and clinical characteristics of patients with distal renal tubular acidosis (dRTA) caused by *SLC4A1* mutations have not been systematically recorded before. Here, we summarized the *SLC4A1* mutations and clinical characteristics associated with dRTA.

**Methods:**

Database was searched, and the mutations and clinical manifestations of patients were summarized from the relevant articles.

**Results:**

Fifty-three eligible articles involving 169 patients were included and 41 mutations were identified totally. Fifteen mutations involving 100 patients were autosomal dominant inheritance, 21 mutations involving 61 patients were autosomal recessive inheritance. Nephrocalcinosis or kidney stones were found in 72.27%, impairment in renal function in 14.29%, developmental disorders in 61.16%, hematological abnormalities in 33.88%, and muscle weakness in 13.45% of patients. The age of onset was younger (*P* < 0.01), urine pH was higher (*P* < 0.01), and serum potassium was lower (*P* < 0.001) in recessive patients than patients with dominant *SLC4A1* mutations. Autosomal recessive inheritance was more often found in Asian patients (*P* < 0.05).

**Conclusions:**

The children present with metabolic acidosis with high urinary pH, accompanying hypokalemia, hyperchloremia, nephrocalcinosis, growth retardation and hematological abnormalities should be suspected as dRTA and suggested a genetic testing. The patients with recessive dRTA are generally more severely affected than that with dominant *SLC4A1* mutations. Autosomal recessive inheritance was more often found in Asian patients, and more attentions should be paid to the Asian patients.

## Introduction

The *SLC4A1* gene is one of the bicarbonate anion transporters of the solute carrier family 4 (SLC4) gene family (1). This gene encodes anion exchanger member 1 (AE1), which is expressed in both erythrocytes and the acid-secreting *α*- intercalated cells of the kidney (2). The erythroid isoform of AE1(eAE1) is expressed in the red blood cells. The kidney anion exchanger 1 (kAE1) is highly expressed in the kidney and located in the basolateral membrane of the *α*-intercalated cell (3). The kAE1 protein plays an essential role in bicarbonate ion (HCO3^−^) in exchange with chloride ion (Cl^−^) during the apical secretion of hydrogen ion (H^+^) into the tubular lumen (4–6).

Mutations in SCL4A1 may cause distal renal tubular acidosis (dRTA). Distal renal tubular acidosis is a disease of defective urinary acidification characterized by impaired H+ secretion into the urine leading to metabolic acidosis, hypokalemia, nephrocalcinosis, and failure to thrive (7, 8). The mutations in *SLC4A1* have been reported in both autosomal dominant (AD) and recessive (AR) types of dRTA. Bruce et al. first reported that dominant dRTA was attributed to *SLC4A1* gene defect in 1997 (9). Mutations associated with AR types of dRTA have usually been found in homozygous and compound heterozygous conditions (10–14).

Our understanding of dRTA caused by *SLC4A1* mutations is limited by low incidence and phenotypic variability, and mutations in *SLC4A1* have not been systematicly documented. However, diagnosis and treatment are often delayed due to clinical variability of the disease, and hereditary kidney disease compromises the quality of life of patients. Herein we analyzed the genetic defects in *SLC4A1* and clinical phenotypes of the patients to facilitate the diagnosis and treatment of dRTA with *SLC4A1* mutations.

## Subjects and methods

### Search strategy and study selection

PubMed, Embase, Web of Science, the China National Knowledge Infrastructure, and Wanfang were searched from the date of inception to May 30, 2022 with the following search terms: “*SLC4A1*” OR “AE1” OR “Band 3”. We also scanned the references of included studies to avoid omissions in the search process. The flow diagram of the search process is provided in Supplementary Figure S1. The protocol for the systmetic review is registered with International Platform of Registered Systematic Review and Meta-analysis Protocols (INPLASY2022120031).

Eligible articles meeting the following criteria were included: (1) patients were diagnosed as dRTA. (2) mutations in *SLC4A1* were confirmed using molecular genetic techniques. (3) clinical data of patients were described. The articles involving a series of patients but without detailed descriptions were excluded.

### Data extraction

From relevant articles meeting the inclusion criteria, the following data of patients were extracted: (1) country, (2) gender, (3) age of onset, (4) mutation information, (5) clinical manifestations, (6) laboratory test results at diagnosis.

### Statistical analyses

The epidemiological and clinical characteristics, and laboratory indexes of patients were described utilizing simple summary statistics. Mann-Whitney *U* test and *t*-test were used to analyze the data. The significance level was set as *p* < 0.05. Statistical analysis was performed using the Statistical Package for the Social Sciences version 26 for Windows (SPSS). Since certain data in some patients were missing, the total number of patients was mentioned in each analysis.

## Results

### Epidemiological characteristics

**Figure 1:**
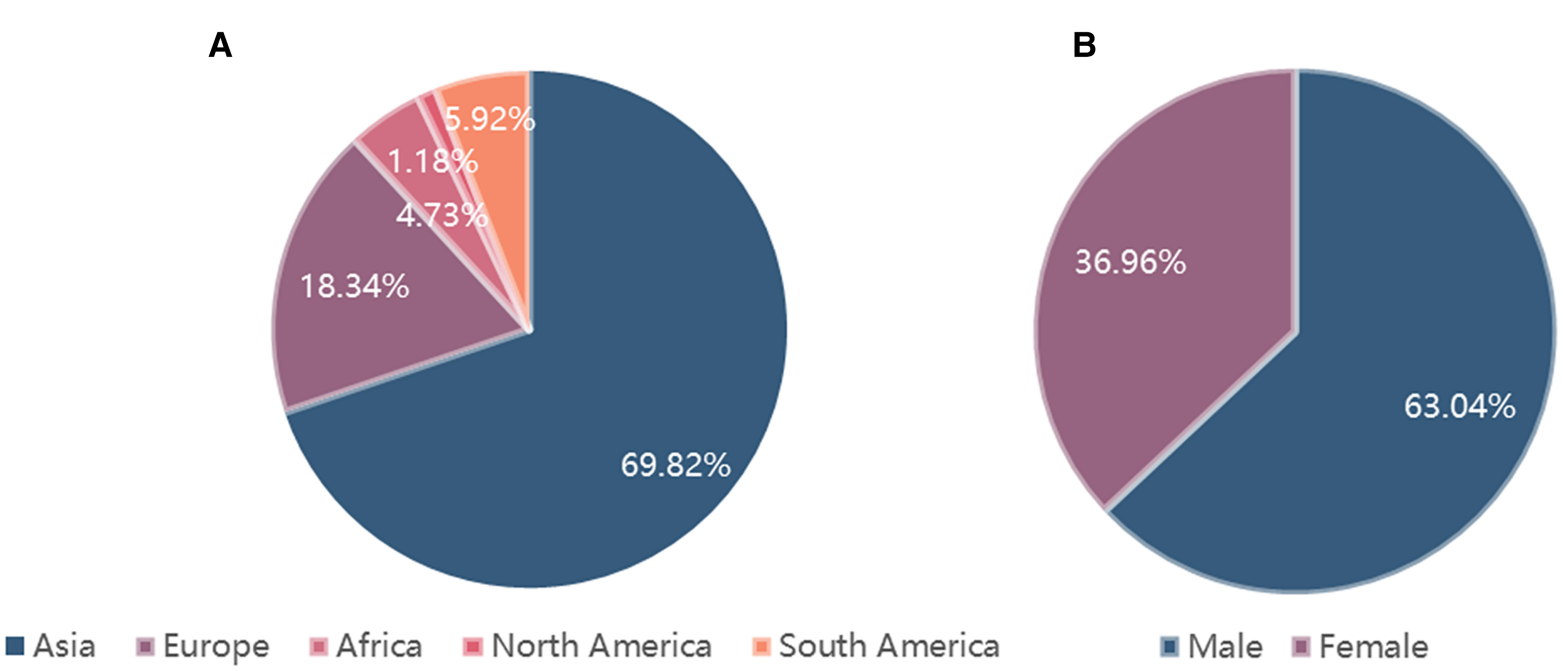
The detailed information of geographical country distribution and gender distribution. (**A**). Geographical country distribution; (**B**). Gender distribution.

The detailed information of geographical country distribution and gender distribution is described in Figure 1. A total of 724 citations were identified through database searches and other resources, and 392 remained after duplicate removal. Fifty-three eligible articles were included ultimately, which contained 169 patients. Among them, Asian cases accounted for the largest part (118/169, 69.82%), followed by European (31/169, 18.34%), South American (10/169, 5.92%), African (8/169, 4.73%), and North American (2/169, 1.18%). Among the Asian, cases from China and Thailand accounted for 37.87% (64/169) and 14.20% (24/169) respectively. Gender distribution was female 36.96% (51/138) vs. male 63.04% (87/138).

### Mutations in *SLC4A1*

**Figure 2:**
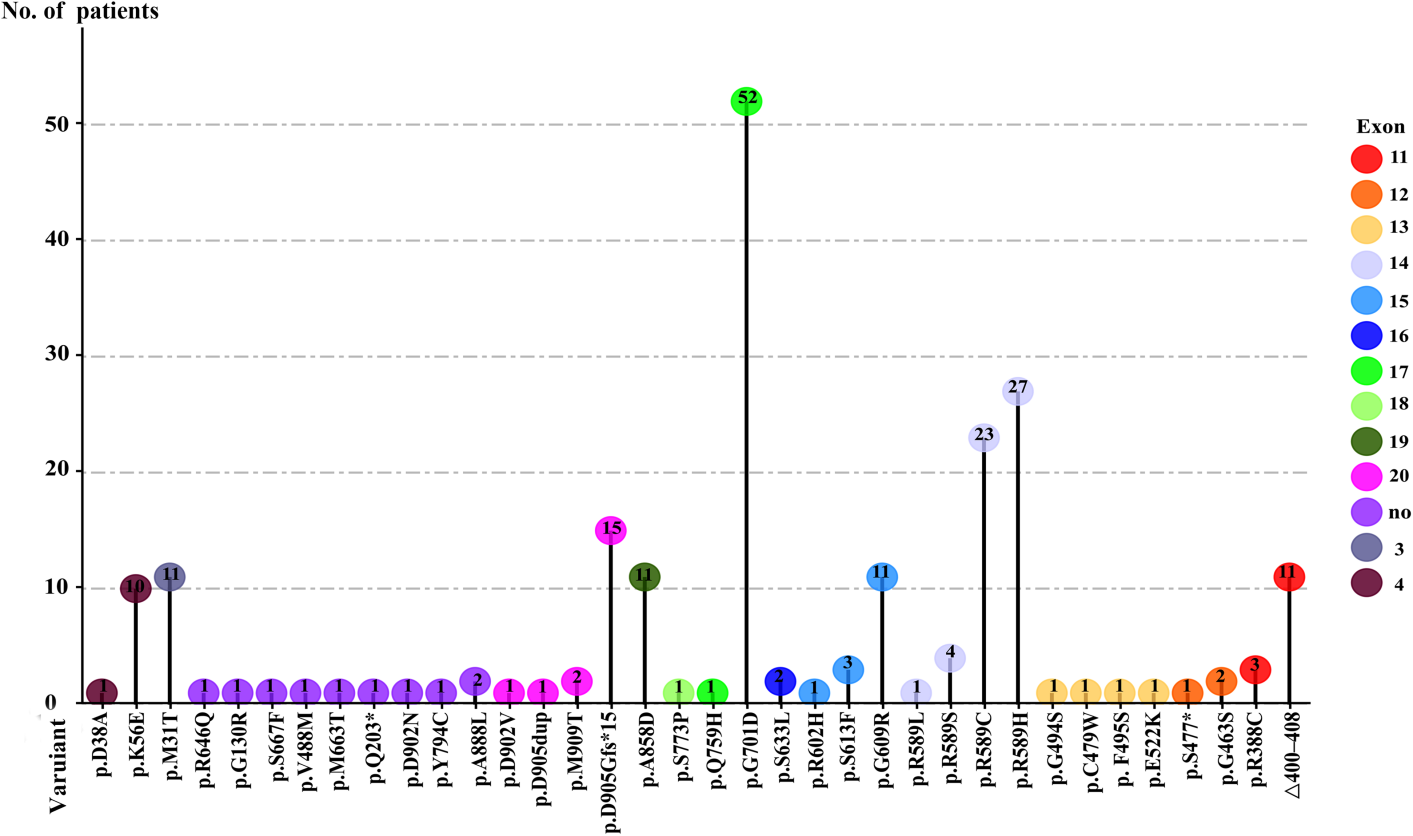
Frenquency of mutations at each mutation site.

**Figure 3:**
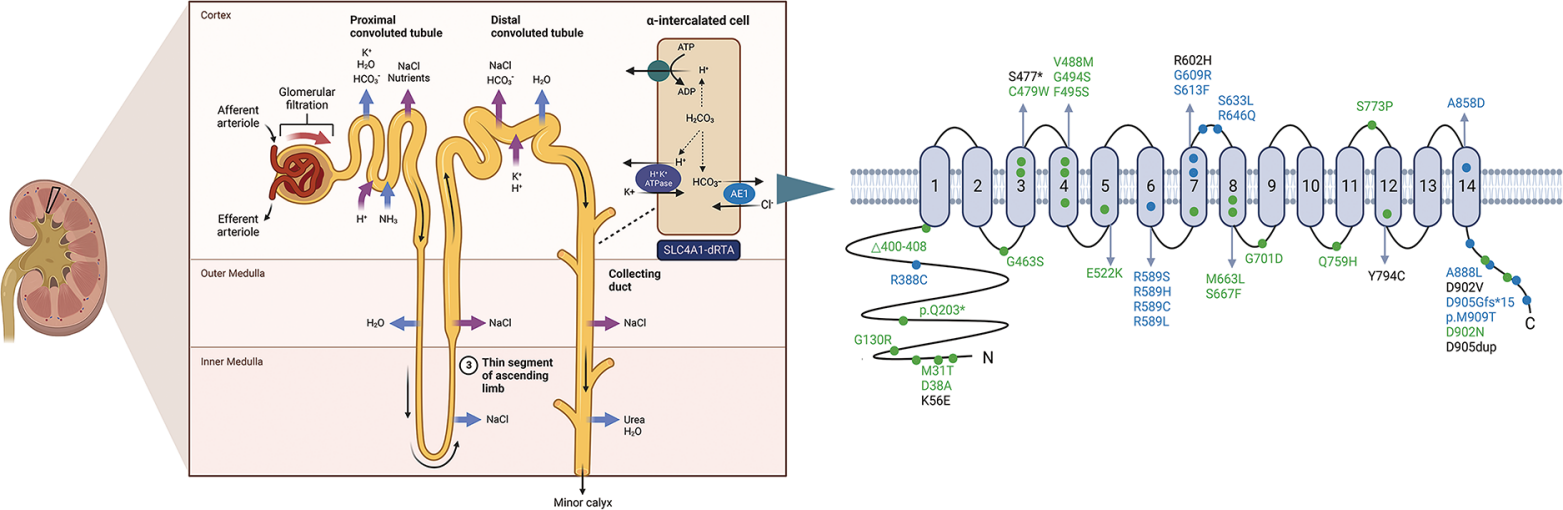
Ae1 protein and the location of the variants. Blue and green dots indicate dominant and recessive mutations, respectively. Black dots indicate the heredity is unknown. Created with Biorender.com.

Mutations in *SLC4A1* are illustrated in Table 1 and Figure 2, and the detailed analytic data of mutation were listed in the Supplementary Table S1. Figure 3 shows the AE1 protein and the location of the variants. Totally 41 mutations were identified in 169 patients. Fifteen mutations involving 100 patients were autosomal dominant inheritance, 21 mutations involving 61 patients were autosomal recessive inheritance. Among the AR mutations, 5 mutations involving 28 patients were autosomal recessive inheritance, and 22 mutations involving 33 patients were compound heterozygosity. The hereditary mode of 7 mutations involving 8 patients were unknown. Thirty-eight patients involving 8 mutations were complicated with Southeast Asian ovalocytosis (SAO), and seven patients involving 7 mutations were complicated with hereditary spherocytosis (HS). The pattern of inheritance was unknown in 6 mutations involving 7 patients. The mutations in Arg589 were the most frequently observed in AD mutations and the most common in all mutations. G701D was the most common in AR mutations. Among 101 Asian patients, 62 patients are autosomal dominant inheritance and 49 patients are autosomal recessive inheritance. Among 51 Non-Asian patients, 39 patients are autosomal dominant inheritance and 12 patients are autosomal recessive inheritance. Autosomal recessive inheritance was more often found in Asian patients (*P* < 0.05).

**Table 1:** Mutations in *SLC4A1*.

No.	Exon	cDNA	Protein
1	14	c.1765C>A	p.R589S
2	19	c.2573C>A	p.A858D
3		NA	p.A888L
4	20	c.2705A>T	p.D902V
5	20	c.2713dupG	p.D905Gfs*15
6	15	c.1981C>T	p.G609R
7	15	c.1825G>A	p.G609R
8	20	c.2840T>C	p.M909T
9	11	c.1162C>T	p.R388C
10	14	c.1766G>T	p.R589L
11	14	c.1922C>T	p.R589C
12	14	c.1765C>T	p.R589C
13	14	c.1766G>A	p.R589H
14		c.1937G>A	p.R646Q
15	15	c.1838C>T	p.S613F
16		c.388G>A	p.G130R
17	17	c.2102G>A	p.G701D
18		c.200°C>T	p.S667F
19			p.V488M
20	17/12/3	c. 2102G>A; c. 1387G>A; c. 92T>C	p.G701D; p.G463S; p.M31T
21	17/3	c. 2102G>A; c. 92T>C	p.G701D; p.M31T
22	14/16	c. 1765C>T; c. 1898C>T	p.R589C; p.S633L
23	17/13	c.2102G>A; c.1480G>A	p.G701D; p.G494S
24	17/13	c.2102G>A; c.1484T>C	p.G701D; p. F495S
25	17/3/4		p.G701D; p.M31T; p.K56E
26	17/18		p.G701D; p.S773P
27		c.2102G>A; c.92T>C; c.694 + 1G>A	
28		c.1199_1225del; c.2102G>A	
29	13/17		p.C479W; p.G701D
30	13/17		p.E522K; p.G701D
31		c.2102 G>A; c.1988T>C	p.G701D; p.M663T
32		c.G2102A; c.607C>T	p.G701D; p.Q203*
33	4/4/17/11		p.D38A; P.K56E; p.Q759H; △400–408
34		c.2102G>A; c.2573C>A	p.G701D; p.A858D
35	17/4/11		p.G701D; K56E; △400–408
36	4/3/17/11		p.K56E; p.M31T; p.G701D; △400–408
37	17/11		p.G701D/△400–408
38	15		p.R602H; △400–408
39		c.2704G>A	p.D902N
40	20	c.2715_2717dupCGA	p.D905dup
41	4	c.166A>G	p.K56E
42	12	c.143°C>A	p.S477*
43		c.2381A>G	p.Y794C
44	20	c.2717_2718ins CGA	
45		c.1199_1225del	

No.	Heredity	Variant state	No. of patients	Associated disease
1	AD	Heterozygous	4	dRTA
2	AD	Heterozygous	9	dRTA
3	AD	Heterozygous	2	dRTA
4	AD	Heterozygous	1	dRTA
5	AD	Heterozygous	15	dRTA
6	AD	Heterozygous	3	dRTA
7	AD	Heterozygous	8	dRTA
8	AD	Heterozygous	2	dRTA
9	AD	Heterozygous	3	dRTA
10	AD	Heterozygous	1	dRTA
11	AD	Heterozygous	17	dRTA
12	AD	Heterozygous	4	dRTA
13	AD	Heterozygous	27	dRTA
14	AD	Heterozygous	1	dRTA
15	AD	Heterozygous	3	dRTA
16	AR	Homozygous	1	dRTA
17	AR	Homozygous	24	dRTA; dRTA/SAO
18	AR	Homozygous	1	dRTA/HS
19	AR	Homozygous	1	dRTA/HS
20	AR	Compound heterozygosity	2	dRTA
21	AR	Compound heterozygosity	7	dRTA
22	AR	Compound heterozygosity	2	dRTA
23	AR	Compound heterozygosity	1	dRTA
24	AR	Compound heterozygosity	1	dRTA
25	AR	Compound heterozygosity	1	dRTA
26	AR	Compound heterozygosity	1	dRTA
27	AR	Compound heterozygosity	1	dRTA
28	AR	Compound heterozygosity	1	dRTA
29	AR	Compound heterozygosity	1	dRTA/HS
30	AR	Compound heterozygosity	1	dRTA/HS
31	AR	Compound heterozygosity	1	dRTA/HS
32	AR	Compound heterozygosity	1	dRTA/HS
33	AR	Compound heterozygosity	1	dRTA/SAO
34	AR	Compound heterozygosity	2	dRTA/SAO
35	AR	Compound heterozygosity	5	dRTA/SAO
36	AR	Compound heterozygosity	1	dRTA/SAO
37	AR	Compound heterozygosity	3	dRTA/SAO
38	AR	Compound heterozygosity	1	dRTA/SAO
39			1	dRTA
40			1	dRTA
41			2	dRTA
42			1	dRTA
43			1	dRTA
44			1	dRTA
45			1	dRTA/SAO

### Clinical characteristics

The age of onset ranged from 0 to 72 years among 129 patients (median 5 years, IQR 2–12). The mean age of onset in patients with autosomal dominant mutations (82 patients), autosomal recessive mutations (22 patients) and compound heterozygosity (21 patients) was 16.33, 6.36 and 4.29 years, respectively. The clinical manifestations of patients diagnosed as dRTA with mutations in *SLC4A1* were described in 121 patients (Table 2). Nephrocalcinosis and kidney stones were presented in 65 (53.72%) and 23 (19.01%) patients respectively, and impairment in renal function was found in 17 (14.29%) patients. Developmental disorders were found in 74 (61.16%) patients, including growth retardation (31.40%) and rickets (29.75%). Hematological abnormalities were observed in 41 (33.88%) patients, including spherocytosis, ovalocytosis and anemia. Muscle weakness was observed in 16 (13.45%) patients. Alkaline urine (PH > 6.5) was presented in 64 (64/79, 81.01%) patients, and the median urine PH in 79 patients was 7.00 (IQR 6.7–7.5). The median blood PH was 7.27 (IQR 7.25–7.30). Hypokalemia was seen in 72 (72/109, 66.06%) patients, and the mean of serum potassium in 109 patients was 3.16 mmol/L. Hyperchloremia was seen in 50 (50/53, 94.34%) patients, and the mean of serum potassium in 109 patients was 113.02 mmol/L.

**Table 2:** The clinical manifestations of patients diagnosed as dRTA with mutations in *SLC4A1*.

Symtoms & signs	Enrolled cases	Prevalence (%)
Nephrocalcinosis	65	53.72%
Kidney stones	23	19.01%
Developmental disorders	74	61.16%
Hematological abnormalities	41	33.88%
Renal dysfunction	17	14.29%
Muscle weakness	16	13.45%
Gastrointestinal symptoms	12	10.08%

**Figure 4:**
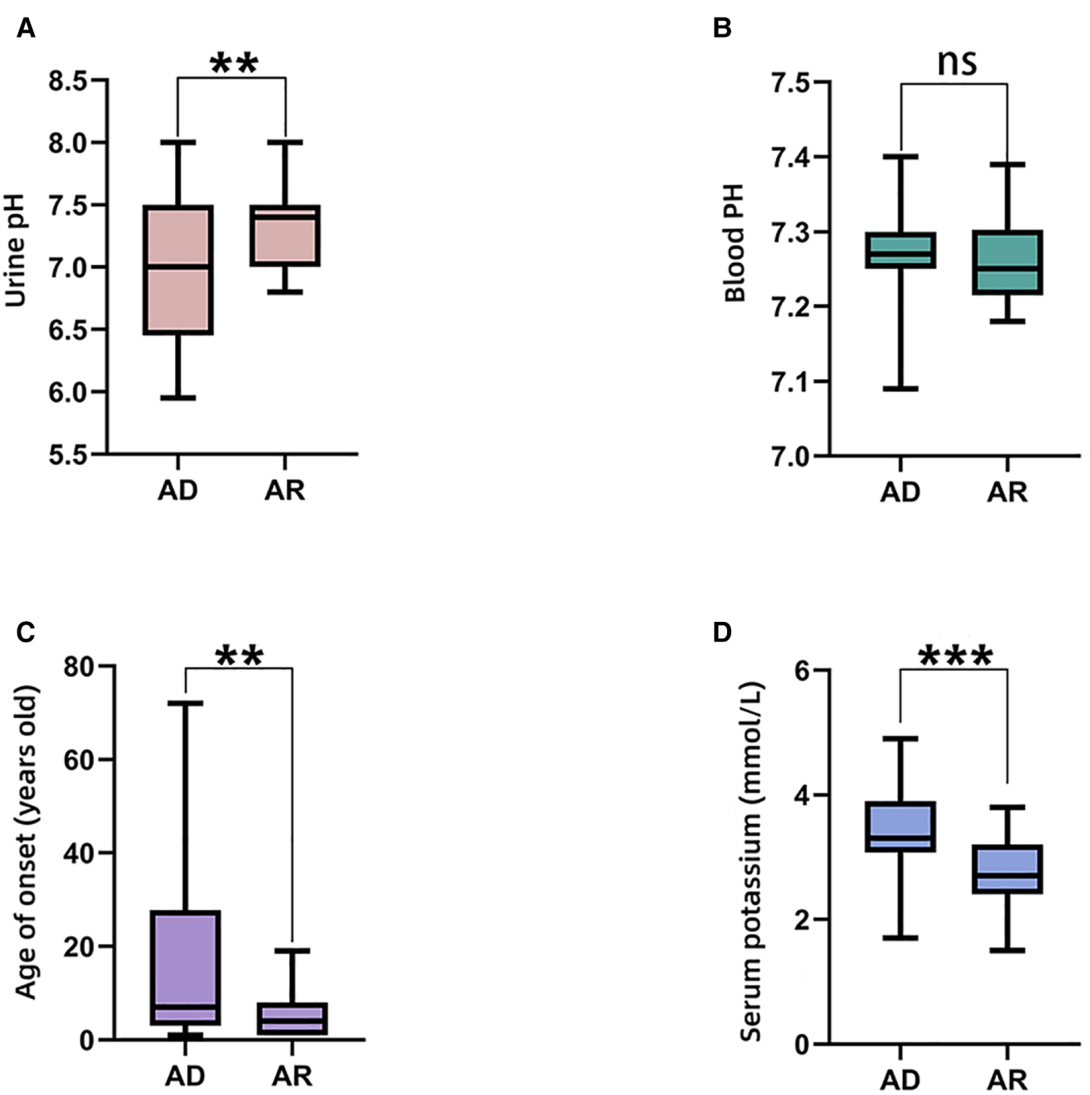
The comparisons of biochemical indexes and age of onset between AD and AR. (**A**). Urine pH; (**B**). Blood PH; (**C**). Age of onset; (**D**). Serum potassium. *P < 0.05; **P < 0.01; ***P < 0.001; ns: *P* > 0.05.

**Figure 5:**
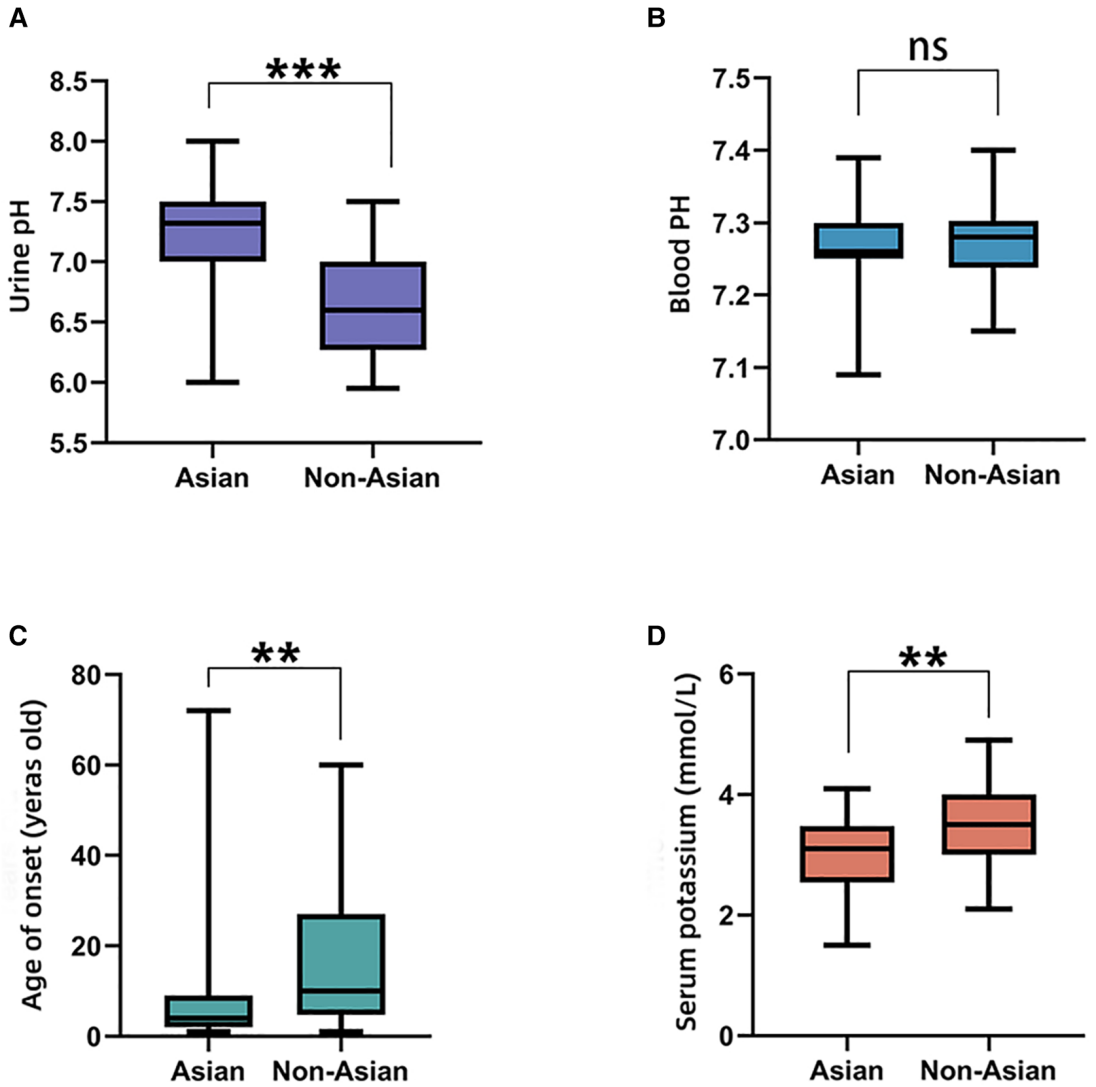
The comparisons of biochemical indexes and age of onset between Asian and Non-Asian. (**A**). Urine pH; (**B**). Blood PH; (**C**). Age of onset; (**D**). Serum potassium. *P < 0.05; **P < 0.01; ***P < 0.001; ns: *P* > 0.05.

**Figure 6:**
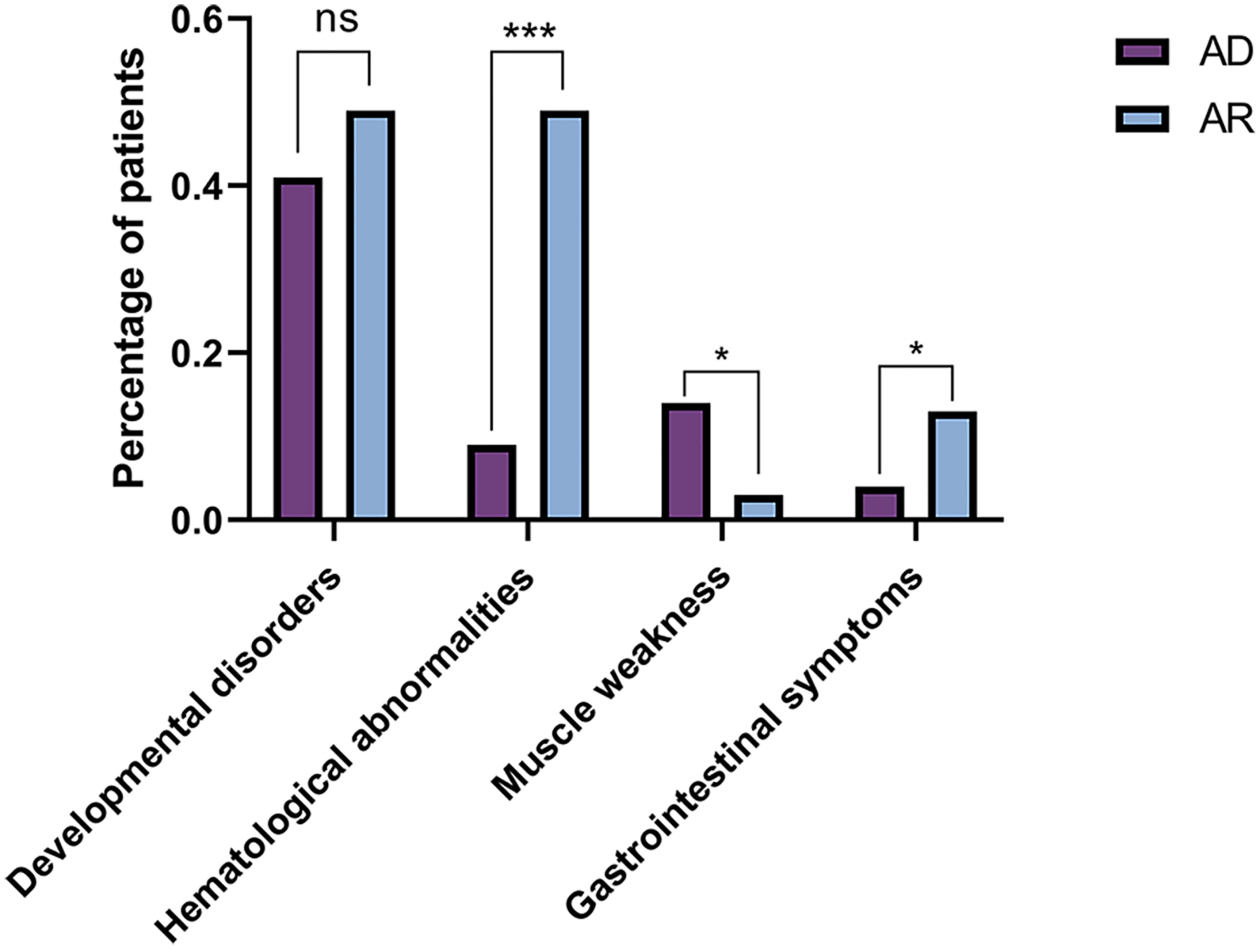
The comparisons of patients involving other organs between AD and AR. *P < 0.05; ***P < 0.001; ns: *P* > 0.05.

The comparisons of biochemical indexes and age of onset between AD and AR *SLC4A1*-dRTA are shown in Figure 4. The age of onset was younger (*P* < 0.01), alkaline urine was more severe (*P* < 0.01), and serum potassium was lower (*P* < 0.001) in recessive patients than patients with dominant *SLC4A1* mutations. Among 61 patients with AR dRTA, hematological abnormalities were observed in 30 (30/61,49.18%) patients, and most of them were from Southeast Asia. However, only 11(10.89%) patients presented with hematological abnormalities in 101 patients with AD dRTA. The comparisons of biochemical indexes and age of onset between Asian and Non-Asian patients are shown in Figure 5. The age of onset was younger (*P* < 0.01), alkaline urine was more severe (*P* < 0.001), and serum potassium was lower (*P* < 0.01) in Asian patients than Non-Asian patients. There are no differences in biochemical indexes and age of onset between male and female (*P* > 0.05). As shown in Figure 6, the recessive forms of dRTA often involves other organs than the kidney, incluing developmental disorders, hematological abnormalities (*P* < 0.001) and gastrointestinal symptoms (*P* < 0.05).

## Discussion

To our knowledge, this is the first study to systematically and comprehensively analyze reported dRTA patients with *SLC4A1* mutations. In this work, we summarized the mutation spectrum and clinical characteristics in patients with *SLC4A1* mutations. *SLC4A1* is located on chromosome 17 and encodes anion exchanger 1 protein in erythrocyte cell membranes and *α*-intercalated cells of the kidney (15).

There are several possible explanations for the pathogenesis of the acid secreting defect secondary to *SLC4A1*. A mouse model lacking AE1 exhibits spontaneous hyperchloremic metabolic acidosis with reduced acid excretion, and inappropriately alkaline urine (16). Basolateral Cl-/HCO3- exchange activity was reduced, while alternate bicarbonate transport pathways were upregulated. Dysregulated expression and localization of the aquaporin-2 water channel were observed in mice lacking AE1, accompanied by severe urinary concentration defect (16). KAE1 forms functional homodimers or etero-oligomers, and oligomerization of a dominant negative mutant AE1 with a wild-type polypeptide in the heterozygote A-IC likely explains some dominant disease. The mutant/wild-type heterodimer fails to traffic to the cell surface or traffics normally but may not function normally at the cell surface (16–18). Dominant dRTA might be caused by inappropriate targeting to A-IC apical membrane, with resultant apical bicarbonate secretion likely short-circuiting luminal acid secretion (19, 20). Whereas, the study by Mumtaz R et al. generated a mouse corresponding to the dominant dRTA mutation in human AE1 R589H, suggesting normal targeting of the pathologic R607H variant, but the mutant mice exhibited reduced expression of V-type ATPase and compromised targeting of this proton pump to the plasma membrane upon acid challenge (21). Homozygous patients may not have any kAE1 isoform at the plasma membrane of a-intercalated cells in the distal nephron since these cells lack glycophorin A and develop dRTA consequently (17). Previous study has also reported that the integrity of the cytosolic COOH terminus played a crucial role in normal kAE1 targeting to the cell surface. Truncation of the last 11 amino acids increased the kAE1 endocytosis rate and reduced recycle to the plasma membrane. The decreased abundance at the plasma membrane and altered recycling provide a possible physiological mechanism of distal renal tubular acidosis in patients carrying *SLC4A1* mutations (22).

The defect of kAE1 is associated with inherited distal renal tubular acidosis (dRTA) (9, 23–26). In addition, eAE1 is an important structural component of the red cell membrane, which ensures the normal skeleton structure and ion transport of erythrocytes (27). Consequently, *SLC4A1* mutations that alter AE1 composition are often associated with disorders of red cell membrane integrity including SAO and HS, which are usually autosomal recessive inheritance (28, 29). SAO is a morphological erythrocyte abnormality caused by a mutational deletion of 27 bp in exon 11 of *SLC4A1*, resulting in a frame 9 amino acid deletion at the junction between the N-terminal domain of eAE1 and the first transmembrane segment, involving codons 400–408 (30). These patients are usually homozygous for a single *SLC4A1* mutation or compound heterozygotes of two different *SLC4A1* mutations found throughout Southeast Asia most commonly, one of which is usually the SAO mutation.

*SLC4A1*-dRTA exhibits different behaviors with regard to different ethnicities. The G701D mutation was first identified in a Thai family (11), which was recessive and frequently observed in Southeast Asia in both homozygote and compound heterozygote with SAO mutation (31). These patients usually accompanied with anemia, jaundice, and splenomegaly; spherocytosis and ovalocytosis could be found in in blood smear (14, 32). Our study showed that almost half of the patients with AR dRTA had hematological abnormalities, while it was uncommon in patients with AD dRTA. Dominant mutant proteins tend to maintain normal Cl-/HCO3- exchange function in erythrocytes, hemolytic anemia was rarely observed consequently (9). The Arg589 is a hotspot mutation site, which is located in the intracellular domain between the sixth and the seventh transmembrane regions of the AE1 protein, more commonly seen in Caucasian (33–35). Even though SAO and G701D are more common in Southeast Asians (10, 14, 31). Since Southeast Asia historically have had a high incidence of Plasmodium falciparum malaria, a presumable supposition is that SAO is considered to have evolved, which might provide a protection against the clinical effects of Plasmodium falciparum malaria (36, 37).

Our study showed that the age of onset varies from birth to adulthood, mostly in childhood. Common clinical manifestations include vomit, failure to thrive, nephrocalcinosis, nephrolithiasis, rickets and muscle weakness. The spectrum of phenotypic severity in dRTA is wide, ranging from growth impairment to renal function impairment. In dRTA, ɑ-intercalated cells in the collecting duct are unable to secrete H+  ions and acidify urine, and result in metabolic acidosis and hypokalemia. Severe hypokalemia could result in vomit and muscle weakness (12, 26, 38). The metabolic acidosis is mainly buffered by the bone, and bone demineralization leads to failure to thrive and rickets. The excess of calcium in the blood and decreased expression of kidney calcium transporter proteins cause hypercalciuria, which results in nephrocalcinosis and/or nephrolithiasis, eventually leads to renal insufficiency (25, 36). The patients often visit different departments due to various clinical manifestations, therefore clinical workers should raise general awareness of the disease.

Our review of the clinical characteristics of these patients revealed the differences between the dominant and recessive forms of patients with *SLC4A1* mutations. The clinical phenotypes of dRTA caused by *SLC4A1* mutations are of great heterogenity. The percentage of homozygous subjects is greater in the Asian population and for this reason they present a more severe clinical picture. Consistent with already published data, the clinical manifestations and biochemical phenotypes of AD are usually milder compared with that of patients with AR mutations (39). Compared with the patients with autosomal dominant mutations, the patients with autosomal recessive mutations had more severe alkaline urine and hypokalemia, and the age of onset was much younger. Probably because the dominant mutants retain wild-type kAE1 protein intracellularly while the recessive kAE1 mutants do not. The patients with the recessive mutants show more severe trafficking defects (37). Based on the above genotype-phenotype correlations, a molecular diagnosis is necessary due to the implications for treatment, prognosis and family risk. For the patients with AR mutations, more attention needs to be paid.

For patients with dRTA, the clinical management is a critical issue that needs to be taken seriously. Since acid-base homeostasis is essential for normal growth and development, the treatments of dRTA should not be limited to correcting the biochemical abnormalities, but also to preventing disease-related abnormalities such as failure to thrive, growth retardation, rickets, osteoporosis, nephrolithiasis, and nephrocalcinosis (40, 41). Since the progression of nephrocalcinosis may lead to chronic kidney disease and end-stage kidney disease in dRTA patients, prevention of nephrocalcinosis is particularly important (40). As citrate salts can prevent nephrolithiasis, potassium citrate is usually recommended (23, 40).

Our results also suggested over half children presented with growth retardation, which has been a major problem in children with renal tubular acidosis, deserving our concerns. At an early stage, alkali and potassium supplementation could correct metabolic acidosis, regulating acid-base balance and significantly improve growth and skeletal deformities (13, 42). And lifelong treatment is recommended (23). Although calcium and active vitamin D are beneficial in the treatment of osteoporosis and osteomalacia, they promote the formation of kidney stones and should be avoided (43). It was reported that combined conventional alkali supplementation with recombinant human growth hormone (rhGH) therapies might have a beneficial effect on growth in dRTA patients, however, further high-quality clinical studies involving more patients are needed to confirm this observation (44). For patients with erythrocyte membrane disorders, be of benefit in patients with severe and moderate hemolytic anemia, but is not necessary in mild cases generally (45).

Our study has several limitations. First, the number of reported cases is not large enough to permit definite genotype-phenotype correlations. Second, this study might have selective bias, since typical or more severe patients tend to be diagnosed and reported. Additionally, the biochemical indexes were from different laboratory, which might cause a slight deviation. And further high-quality studies are needed for the purpose of explaining the more precise molecular mechanism.

## Conclusion

In summary, our study firstly summarized mutations and clinical characteristics of dRTA caused by *SLC4A1* mutations. The patients with the presence of metabolic acidosis, hypokalemia, hyperchloremia, nephrocalcinosis and growth retardation should be prompt a genetic test as soon as possible. The patients with recessive dRTA are generally more severely affected and the age of onset is earlier than that with dominant *SLC4A1* mutations, and autosomal recessive inheritance was more often found in Asian patients. Early identification and early treatment are very essential for the prognosis of patients, especially the Asian patients.

## Data Availability

The original contributions presented in the study are included in the article/**Supplementary Material**, further inquiries can be directed to the corresponding author/s.
