# Supplementary material for: Mutations and clinical characteristics of dRTA caused by SLC4A1 mutations: Analysis based on published patients
Source: Front Pediatr. 2023 Jan 26;11:1077120. doi: 10.3389/fped.2023.1077120 (PMC9910804; doi:10.3389/fped.2023.1077120)
Supplement: Supplementary file 1 [file Datasheet1.pdf]

Fig S1. The flow diagram of the search process.

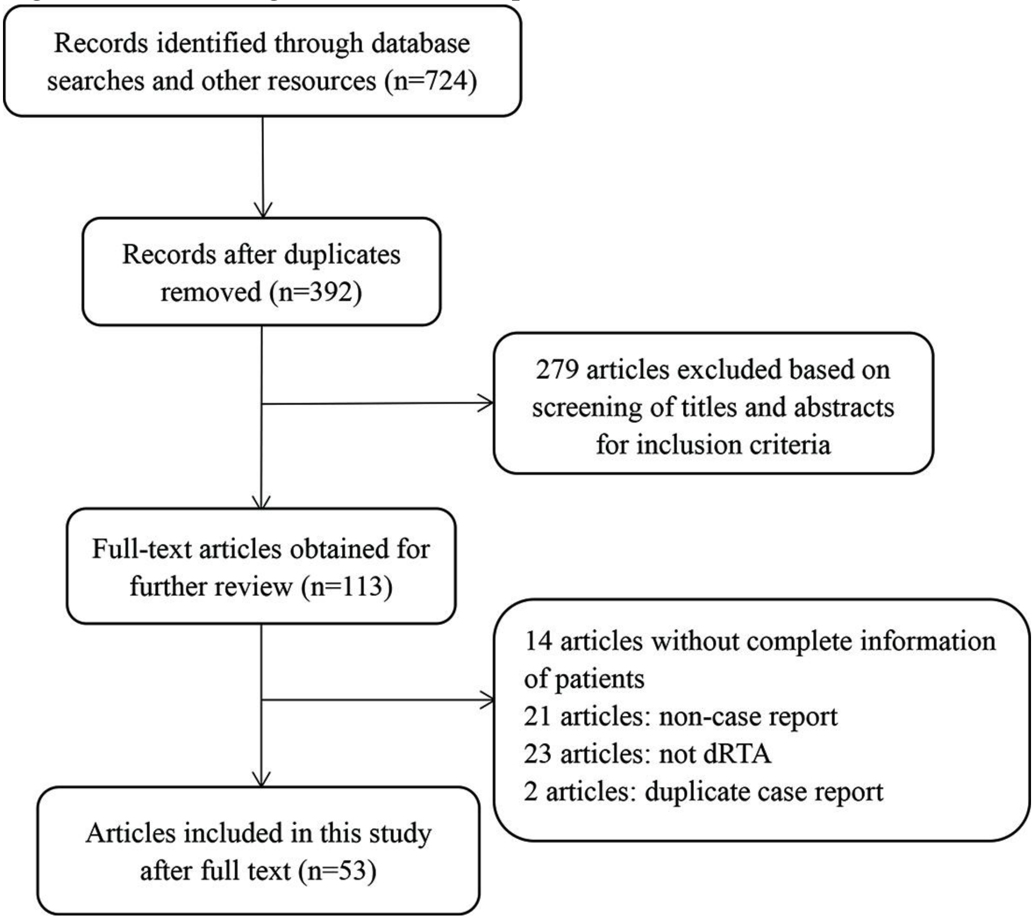

Table S1. The number of patients with mutations at each mutation site.

Exon	Protein	No. of patients
3	p.M31T	11
4	p.K56E	10
	p.D38A	1
11	p.R388C	3
	△400–408	11
12	p.S477*	1
	p.G463S	2
13	p.G494S	1
	p.C479W	1
	p. F495S	1
	p.E522K	1
14	p.R589S	4
	p.R589L	1
	p.R589C	23
	p.R589H	27
15	p.G609R	11
	p.S613F	3
	p.R602H	1
16	p.S633L	2
17	p.G701D	52
	p.Q759H	1
18	p.S773P	1
19	p.A858D	11
20	p.D902V	1
	p.M909T	2
	p.D905Gfs*15	15
	p.D905dup	1
Unknown	p.A888L	2
	p.R646Q	1
	p.G130R	1
	p.S667F	1
	p.V488M	1
	p.M663T	1
	p.Q203*	1
	p.D902N	1
	p.Y794C	1
